# Oligodendrocyte precursor cell transplantation attenuates inflammation after ischemic stroke in mice

**DOI:** 10.3389/fneur.2025.1583982

**Published:** 2025-07-16

**Authors:** Li-Ping Wang, Chang Liu, Yuanyuan Ma, Aijuan Yan, Guo-Yuan Yang, Xinkai Qu, Wenshi Wei

**Affiliations:** ^1^Department of Neurology, Huadong Hospital, Fudan University, Shanghai, China; ^2^Department of Geriatric Medicine, Huadong Hospital, Fudan University, Shanghai, China; ^3^School of Biomedical Engineering and Med-X Research Institute, Shanghai Jiao Tong University, Shanghai, China; ^4^Department of Neurology, Zhongshan Hospital, Fudan University, Shanghai, China

**Keywords:** blood–brain barrier, inflammation, ischemic stroke, oligodendrocyte precursor cell, transplantation

## Abstract

**Background:**

Disruption of blood–brain barrier and neuroinflammation are critical pathological features in the acute phase of ischemic stroke. This study investigates whether oligodendrocyte precursor cell transplantation can downregulate inflammation to attenuate blood–brain barrier disruption following ischemic brain injury.

**Methods:**

Adult male Institute of Cancer Research mice (*n* = 60) underwent transient middle cerebral artery occlusion. Post ischemic assault, these mice received a stereotactic injection of oligodendrocyte precursor cells (6 × 10^5^). Neurobehavioral outcomes, infarct volume, inflammatory cytokines, myeloperoxidase, and tight junction protein levels were measured following ischemia.

**Results:**

Oligodendrocyte precursor cell transplantation reduced infarct volume, alleviated anxiety and depression, and promoted neurological recovery after ischemic stroke. Compared to the control group, oligodendrocyte precursor cell treated mice exhibited reduced levels of inflammatory cytokines IL-1β, IL-6, and TNF-α, reduced neutrophil infiltration, and diminished loss of tight junction protein. Oligodendrocyte precursor cells alleviated inflammation by increasing β-catenin expression. The administration of β-catenin inhibitor blocked the beneficial effects of oligodendrocyte precursor cell transplantation on neuroinflammation and blood–brain barrier permeability.

**Conclusion:**

This study demonstrates that oligodendrocyte precursor cell transplantation attenuates neuroinflammation and protectes blood–brain barrier in the acute phase of ischemic stroke. Our findings indicate that oligodendrocyte precursor cell transplantation is a promising therapeutic approach for ischemic stroke.

## Introduction

Ischemic stroke is one of the leading causes of death and disability in the world (1, 2). Currently, thrombolysis and endovascular interventional therapy are the most effective treatment strategies in the early stage after ischemic stroke, but the time windows for both treatments are limited (3, 4). Many pathological processes, including blood–brain barrier (BBB) dysfunction, inflammation, excitotoxicity, oxidative stress, neuronal loss, and glial activation, are involved in stroke progression (5).

BBB disruption and neuroinflammation are critical pathological features in the acute phase of cerebral ischemia (6, 7). BBB is comprised of brain endothelial cells with their tight junctions, the basement membrane, pericytes, and astrocyte end-feet (8). Brain endothelial cells express high levels of tight junction proteins, which determine BBB integrity (8, 9). After ischemic stroke, inflammatory responses at the blood-endothelial interface of brain capillaries are the basis of ischemic tissue damage (10). Post-ischemic inflammation is associated with acute BBB disruption, vasogenic edema, hemorrhagic transformation, and worse neurological outcomes in animals and humans (11). The proinflammatory signals from immune mediators activate resident cells and influence infiltration of inflammatory cells into the ischemic region, exacerbating brain damage (12). Neutrophils migrate through the endothelial vessel wall and are attracted towards the ischemic area (13). Neutrophils cause secondary injury by releasing proinflammatory factors, reactive oxygen species (ROS), proteases, and matrix metalloproteinases (MMPs) (12). Therefore, targeting neuroinflammation may be a promising therapeutic approach for the treatment of ischemic stroke.

Oligodendrocyte precursor cells (OPCs) are derived from the ventricular zone in the embryo and migrated widely through the central nervous system (14). OPCs could maintain BBB integrity during development and mediate the remyelination process after brain injury (15). OPC transplantation showed a promising potential for ischemic stroke therapy (16–18). In our previous study, OPC transplantation could attenuate tight junction disruption in brain endothelial cells in the acute phase of ischemic stroke and promote angiogenesis and remyelination in the chronic phase of ischemic stroke via activating the Wnt/β-catenin pathway. As neuroinflammation is closely related to BBB integrity, we hypothesize that the protective role of OPC transplantation on BBB is achieved through attenuating neuroinflammation in ischemic stroke.

In this study, we use a mouse model of transient middle cerebral artery occlusion (tMCAO) to explore whether OPC transplantation downregulates inflammation to attenuate BBB disruption after ischemic brain injury.

## Materials and methods

### Experimental protocol

Animal procedures and protocols were approved by the Institutional Animal Care and Use Committee (IACUC) of Fudan University, Shanghai, China. Animal studies were reported according to ARRIVE 2.0 guidelines. Adult male Institute of Cancer Research mice (*n* = 60) weighing 28–30 g (JSJ, Shanghai, China) were used in the study. Animals were housed with free access to water and food. Mice were randomly divided into four groups: sham group, phosphate buffered saline (PBS) treated group, OPC-treated group, and OPC-treated plus β-catenin inhibitor (XAV-939) group, *n* = 10–16 per group.

### OPC isolation and identification

The brain cortex was dissected from P1 Sprague–Dawley rat pups as described (19, 20). Brain tissue was dissociated into a single-cell suspension and was filtered with a 70-μm filter. Then cells were seeded on poly-d-lysine (PDL, Sigma, St. Louis, MO) coated culture flasks in DMEM (Gibco, Carlsbad, CA) with 10% fetal calf serum (Gibco). Eight days later, the microglia were separated from glia cell mixtures after 30 min of culture by a 220-rpm shake and then OPCs were collected by 20 h of culture by a 200-rpm shake. Collected cells were injected into the mouse or seeded on a PDL-coated culture dish in Neurobasal-A (Gibco) containing 2% B27 (Gibco), 10 ng/mL PDGF-AA (Gibco), 10 ng/mL bFGF (Peprotech, Rocky Hill, NJ) and 2 mmoL/L glutamine (Gibco).

For identification, OPCs were incubated with primary antibodies against NG2 (1:200, Millipore, Bedford, MA), GAFP (1:200, Millipore), MBP (1:200, Abcam, Cambridge, United Kingdom), NeuN (1:200, Millipore), and Iba-1 (1:200, WAKO, Osaka, Japan) at 4°C overnight. Then cells were incubated with the fluorescence conjugated second antibodies at 37°C for 1 h.

### Establishing a mouse model of tMCAO

The mouse model of tMCAO was performed as described previously (21). Mice were anesthetized with 1.5% isoflurane (RWD Life Science, Shenzhen, China) and placed supine. After the isolation of the left common carotid artery, external carotid artery, and internal carotid artery, the origin of the middle cerebral artery was occluded by a silicone-coated 6-0 suture (Covidien, Mansfield, MA). The suture was withdrawn after 90 min of tMCAO. The success of occlusion was assessed by the laser Doppler flowmetry (Moor Instruments, Devon, United Kingdom) with a decrease of cerebral blood flow at least 80% of the baseline. Sham mice were conducted in the same procedure except for the insertion of suture.

### Transplantation of OPCs

**Figure 1:**
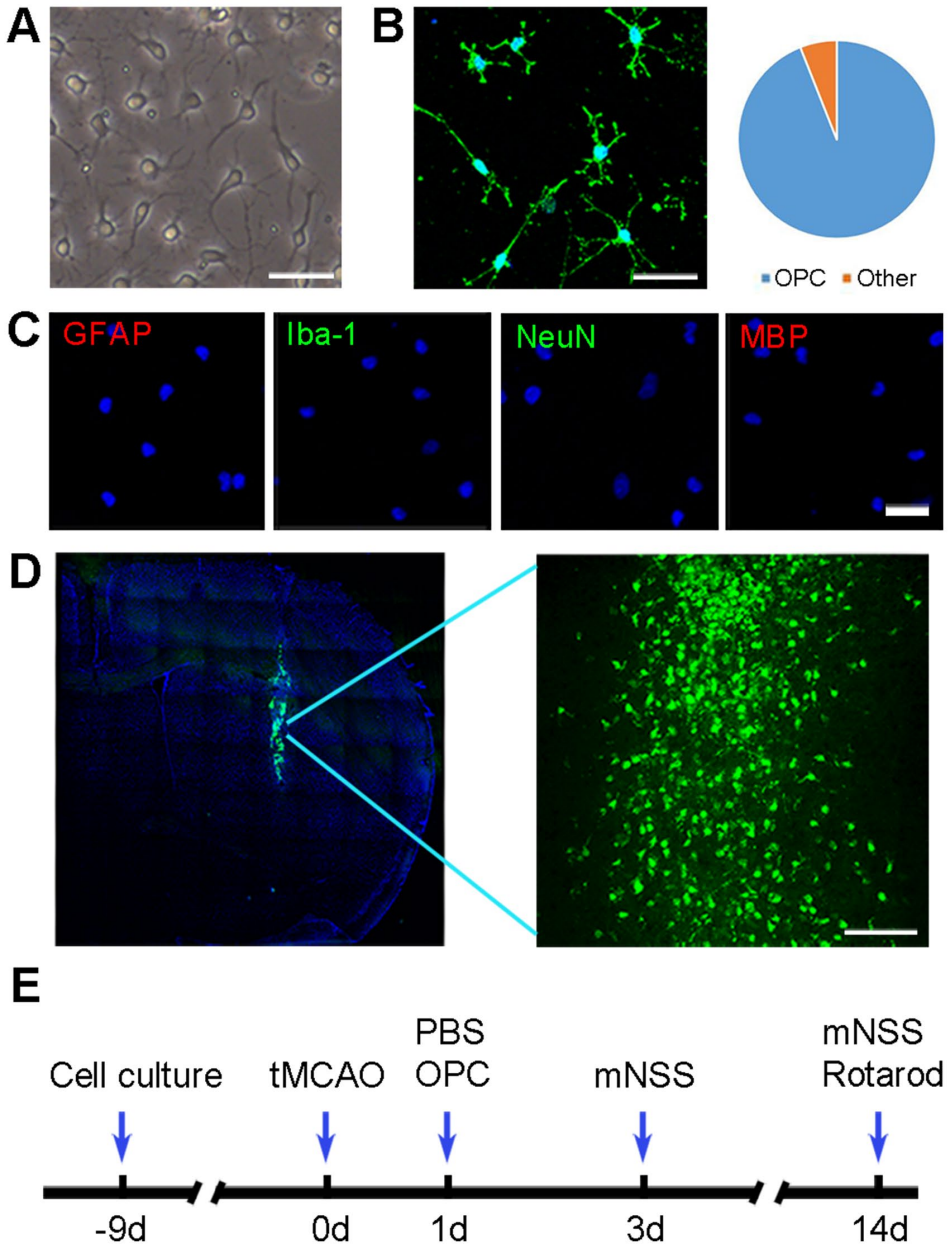
OPC identification and transplantation. **(A)** Morphology of cultured cells under phase-contrast microscopy. Scale bar = 50 μm. **(B)** Representative image of NG2 in cultured cells. Scale bar = 50 μm. **(C)** Immunofluorescence staining showed that cultured cells were negative for GFAP, Iba-1, NeuN, and MBP. Scale bar = 50 μm. **(D)** Green fluorescent OPCs (CFDA-SE stained) were located in the ischemic hemisphere. Scale bar = 100 μm. **(E)** Diagram of experimental design.

OPCs were injected at 24 h after tMCAO (Figure 1E). Before transplantation, OPCs were labeled with carboxyfluorescein diacetate-succinimidyl ester (CFDA-SE, Beyotime, Shanghai, China) for tracking. The mice after tMCAO were anesthetized and received stereotaxic transplantation. A microsurgical drill made a small skull hole 2 mm lateral to the bregma. An amount of 6 × 10^5^ OPCs was suspected in 5 μL PBS and slowly injected into the left striatum at 2 mm lateral to the bregma and 3 mm under the dura (AP = 0 mm, ML = 2 mm, DV = 3 mm) at a rate of 1 μL/min by the 10 μL Hamilton syringe (Hamilton, Bonaduz, Switzerland) (17, 18, 22, 23). The same amount of PBS was injected as control (16).

### Administration of drugs

The mice were injected i.p. daily with cyclosporine A (5 mg/kg, Sigma) for immunosuppression after cell transplantation. The PBS group and sham group were administered cyclosporine A as well. For the OPC plus XAV-939 group, the β-catenin inhibitor XAV-939 (40 mg/kg, MCE, Monmouth Junction, NJ) was injected intraperitoneally once a day (16, 24).

### Neurobehavioral assessment

The modified neurological severity score (mNSS) was performed by an investigator who was blinded to the experimental treatment to evaluate the neurological function at 3 and 14 days after tMCAO. The mNSS ranged from 0 to 14 and included motor, sensory, balance, and reflex tests (25).

Rotarod test was performed at 7 and 14 days after tMCAO to test the motor coordination and balance. Briefly, Mice were trained on a rotating rod at 20 rpm for 3 consecutive days before tMCAO. Mice were placed on the rod for adaption, after which the rod was continuously accelerated to 40 rpm. The mice were monitored, and the time mice stayed on the rod (latency to fall) was recorded (17).

The novel object recognition test is used to test cognition behavior. The apparatus is a box 45 cm × 45 cm × 45 cm in size. The first 3 days are for adaption. Each mouse was placed in the apparatus and allowed to explore freely for 10 min. On the inspection day, two objects of the same shape, size, and color are placed on the bottom of the box. Each mouse is placed inside the instrument and allowed to explore freely for 10 min. One hour later, one of the objects is replaced by a novel object with different shapes and colors. Each mouse is put back into the apparatus and allowed to explore freely for 5 min. The novel object preference index is time spent exploring novel objects/total time to explore novel and familiar objects (26, 27).

The tail suspension test is a test for the antidepressant activity (17). In the test, the tail of the mouse is suspended on a lever with tape, and a camera is used to record its behavior. The mouse struggles to escape for a period of time and then adopt a posture of immobility. After 2 min of induction time, the time for each mouse to keep immobility and the total time are recorded within left 4 min. Depression can decrease the frequency and duration of locomotor activity.

The elevated plus maze test is used to evaluate anxiety-related behaviors which is based on the test animals’ aversion to open spaces when feeling anxious (20). The equipment consists of a “+”-shaped maze elevated above the ground with 2 opposite closed arms, 2 opposite open arms, and a central area. Choice behavior was observed for 10 min. The number of entries to the open arms were counted and the time in the open arms were recorded by a video camera installed above the maze.

### Brain infarct volume measurement

Mice were sacrificed at 3 days after tMCAO, and brains were cut into a series of 20 μm thick coronal sections. The cresyl violet staining (Sigma) was performed to measure the brain infarct volume. Infarct volume was calculated using ImageJ software (National Institutes of Health, Bethesda, MD) as described previously (5).

### Immunofluorescence staining

Brain slices were fixed with methanol at 4°C for 10 min and blocked with diluted donkey serum (Jackson ImmunoResearch, West Grove, PA) for 60 min at room temperature. Slides were incubated with primary antibodies of MPO (1:200, R&D system, Minneapolis, MN), Iba-1 (1:200, NB100-1028, Novusbio, CO), CD31 (1:200, R&D system), occludin (1:200, Invitrogen, Carlsbad, CA), ZO-1 (1:100, Invitrogen) overnight at 4°C. After rinsing three times with PBS, brain slices were incubated with the fluorescence conjugated second antibodies for 1 h at room temperature. Immunofluorescence photos were collected by a confocal microscope (Leica, Solms, Germany). We measured the vessel length and gap by ImageJ software (National Institutes of Health). Gap length was presented as a percentage (%) of gap length in the whole vessel (16).

### Real-time PCR analysis

Regional brain tissues from the infarct hemisphere of ischemic mice, including cortex and striatum, were isolated for real-time PCR. The real-time PCR assay was performed as described previously (28). The two-stage RT-PCR amplification parameters were 95°C for 30 s followed by 40 cycles of 95°C for 5 s and 60°C for 30 s. The mRNA expression level was normalized to reference gene GAPDH and displayed as relative expression of mRNA by 2^−ΔΔCt^ method.

### Western blot analysis

Western blot was performed as described previously (17, 29). The membranes were blocked with 5% skim milk and incubated with primary antibodies against β-catenin (1:500, Abcam) and GAPDH (1:1000, Abcam) overnight at 4°C. After washing with TBST buffer, the membranes were incubated with HRP-conjugated secondary antibody.

### Statistical analysis

The sample size was determined according to our previous publications for similar outcomes (17, 20). For immunohistochical images, we adopted the manual fluorescence positive cell counting or software to measure the length of linear expression (21, 22). Analysis was performed by Prizm Graphpad 9. Multiple comparisons were analyzed using one-way ANOVA followed by Tukey’s post-hoc test for normally distributed data. Comparisons between the two groups were carried out using Student’s *t*-test. Data were expressed as mean ± SD. A probability value of less than 0.05 was considered significant.

## Results

### OPC identification and transplantation

Cultured OPCs showed a bipolar or multipolar morphology under phase contrast microscope (Figure 1A). The cultured OPCs were identified by immunofluorescence 10 days after isolating from P1 rat brains. Immunofluorescent staining showed that the percentage of NG2^+^ cells was 94% (Figure 1B). Very few cells expressed GFAP, Iba-1, NeuN or MBP (Figure 1C). Transplanted OPCs were labeled with CFDA-SE for *in vivo* cell tracking. The results demonstrated that a considerable number of transplanted OPCs could survive at 3 days after tMCAO (Figure 1D).

### OPC transplantation reduced infarct volume, alleviated anxiety and depression, and improved neurobehavioral recovery after tMCAO

**Figure 2:**
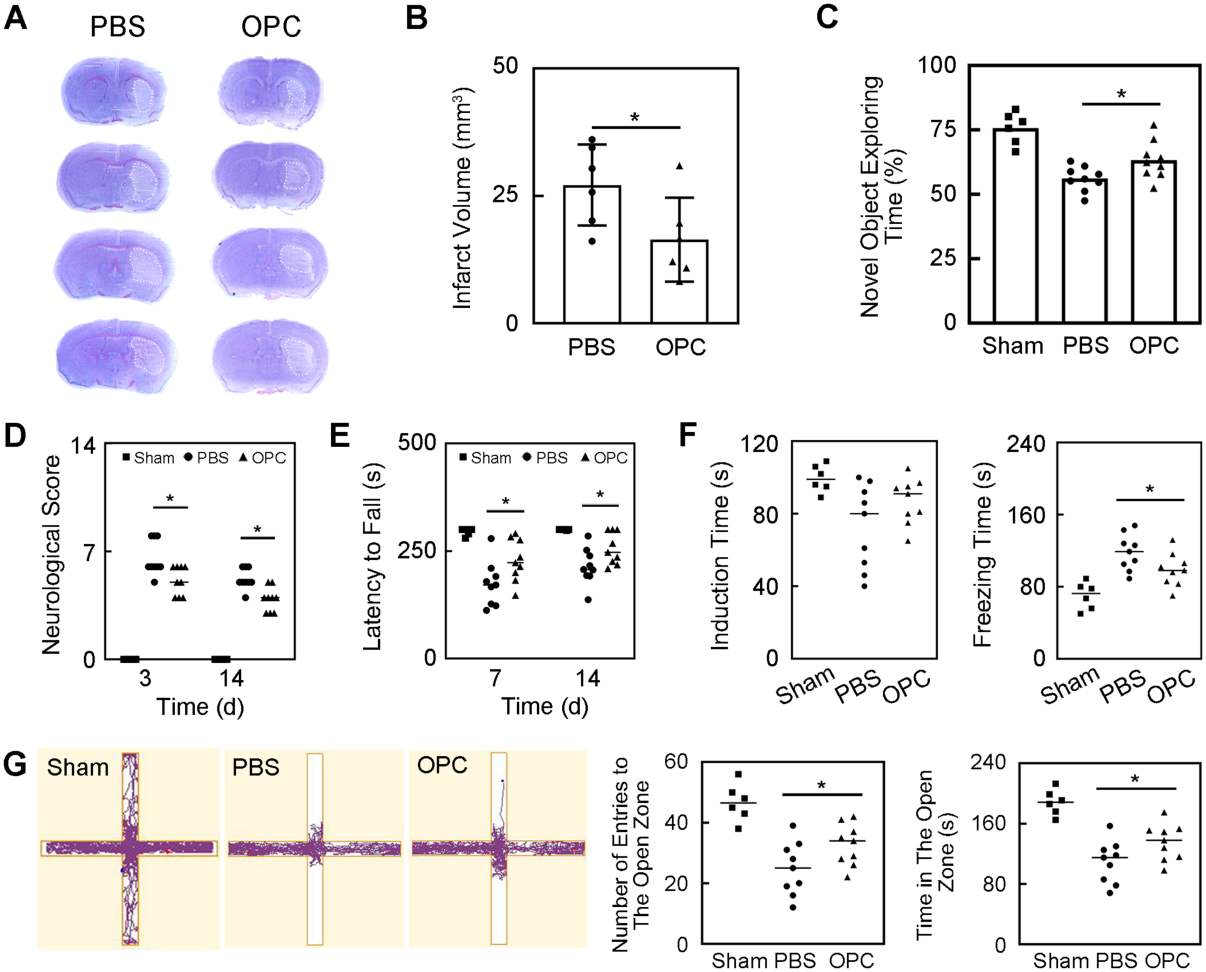
OPC transplantation reduced infarct volume and improved neurobehavioral recovery after tMCAO. **(A)** Cresyl violet staining showed the infarction after tMCAO in PBS and OPC groups. **(B)** Bar graph indicated that OPC transplantation reduced infarct volume. *N* = 6 per group. **(C)** Bar graph of novel object exploring time in the novel object recognition test. *N* = 6–9 per group. **(D)** Bar graph of neurological scores. *N* = 6–9 per group. **(E)** Bar graph of latency to fall. *N* = 6–9 per group. **(F)** Bar graphs of induction time and freezing time in the tail suspension test. *N* = 6–9 per group. **(G)** The track plot and bar graphs of number of entries to the open zone and time in the open zone in the plus maze test. *N* = 6–9 per group. Data are mean ± SD, ^*^p < 0.05.

The brain infarct volume was evaluated using cresyl violet staining. Results showed that infarct volume was significantly decreased in the OPC-treated mice compared to the control (PBS) mice at 3 days after tMCAO (Figures 2A,B, *p* < 0.05).

The novel object recognition test was performed to detect the memory and cognition. OPC transplantation increased the new object exploring time at 14 days after tMCAO which indicating better memory and cognitive abilities (Figure 2C, *p* < 0.05).

The mNSS and rotarod test were performed to evaluate the neurological function. OPC transplantation significantly decreased neurological scores at 3 and 14 days after tMCAO (Figure 2D, *p* < 0.05). The rotarod test demonstrated that the time staying on the rotarod was prolonged in OPC-treated mice compared to control mice at 7 and 14 days after tMCAO (Figure 2E, *p* < 0.05). The OPC-treated group attenuated the neurobehavioral deficiency compared to the control group.

The tail suspension test was performed to assess the depression-like behavior. The induction time and freezing time indicated depression level. The decrease of the induction time or the increase of the freezing time represented that the mice tended to be more depressed. Our test showed that there was no difference in the induction time. The freezing time significantly decreased in OPC-treated mice compared to the PBS-treated mice (Figure 2F, *p* < 0.05). This result indicated that OPC transplantation could alleviate post-stroke depression.

The plus maze test was performed to estimate the anxiety behavior of mice. The number of entries to the open zone and the time in the open zone of OPC-treated mice were increased compared to the control (Figure 2G, *p* < 0.05). This indicated that OPC transplantation could attenuate anxious behavior.

### OPC transplantation downregulated the expression of inflammatory factors after tMCAO

**Figure 3:**
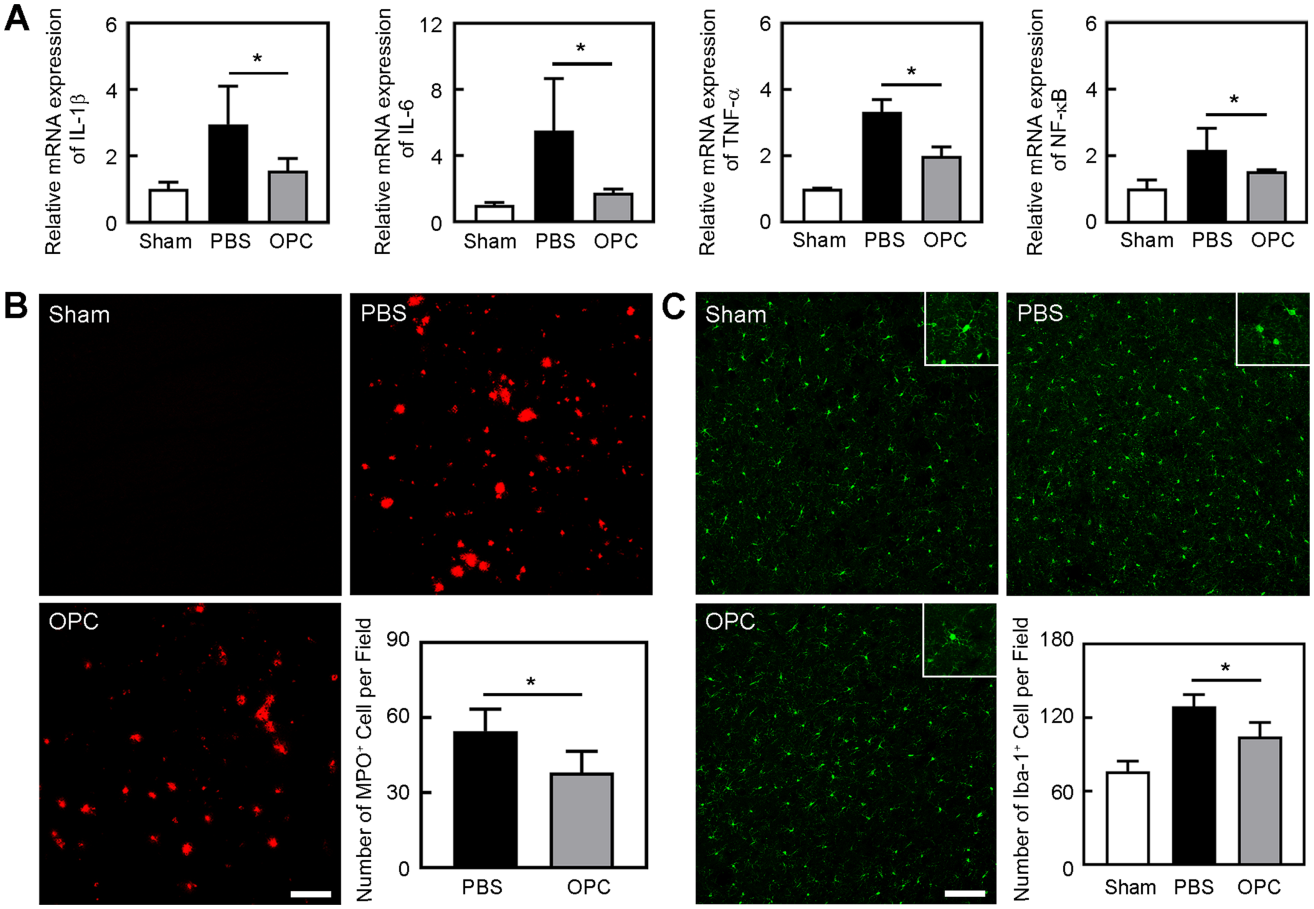
OPC transplantation reduced inflammation after tMCAO. **(A)** Bar graphs showed the mRNA level of IL-1β, IL-6, TNF-α, and NF-κB in the sham, PBS, and OPC groups at 3 days after tMCAO. *N* = 4 per group. **(B)** Representative images of MPO staining in the peri-infarct area at 3 days after tMCAO. Bar graph showed the quantification of MPO^+^ cells. Scale bar = 100 μm. *N* = 6 per group. **(C)** Representative images of Iba-1 staining in the peri-infarct area at 3 days after tMCAO. Bar graph showed the quantification of Iba-1^+^ cells. Scale bar = 100 μm. *N* = 6 per group. Data are mean ± SD, ^*^p < 0.05.

Inflammatory factors IL-1β, IL-6, and TNF-α were upregulated at 3 days after tMCAO. However, OPC transplantation reduced IL-1β, IL-6, and TNF-α expression compared to the control group (Figure 3A, *p* < 0.05). Besides, OPC transplantation downregulated the NF-κB pathway related to inflammatory factors (Figure 3A, *p* < 0.05).

### OPC transplantation alleviated the leukocyte infiltration and increase of microglia after tMCAO

Neutrophils are the primary inflammatory cell type that responds to the inflammatory stimulus following ischemic stroke (30). To determine whether OPC transplantation alleviated leukocyte infiltration after tMCAO, we conducted immunostaining to examine the number of MPO^+^ cells. We found that OPC transplantation reduced the number of MPO^+^ cells in the ipsilateral brain at 3 days after tMCAO (Figure 3B, *p* < 0.05). It was noted that neutrophil infiltration was greatly reduced in the OPC-transplanted mice after tMCAO.

Microglia are resident immune cells in the central nervous system. To detect whether OPC transplantation affected the number of microglia, we conducted immunostaining to examine the number of Iba-1^+^ cells. We found that OPC transplantation reduced the number of Iba-1^+^ cells in the ipsilateral brain at 3 days after tMCAO (Figure 3C, *p* < 0.05). So, OPC transplantation alleviated the increase of microglia after tMCAO.

### OPC transplantation attenuated BBB disruption after tMCAO

**Figure 4:**
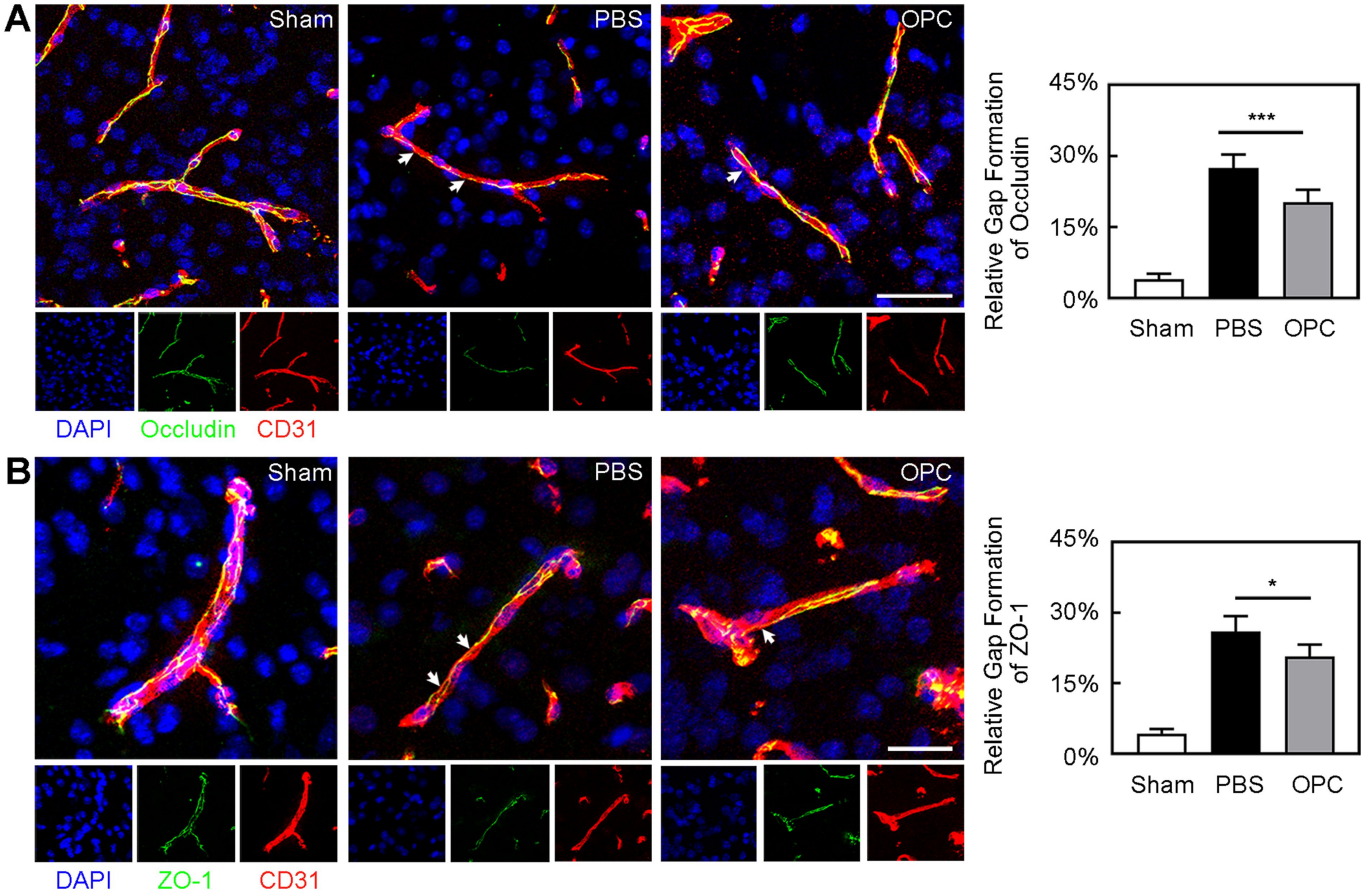
OPC transplantation attenuated the breakage of occludin and ZO-1 after tMCAO. **(A)** Three-dimension reconstruction confocal microscopy images of occludin (green), endothelial marker CD31 (red) and DAPI (blue) at 3 days after tMCAO in three groups. The white arrows indicated the gap formation of occludin. Bar graph showed the quantification of gap formation of occludin. Scale bar = 50 μm. **(B)** Three-dimension reconstruction confocal microscopy images of ZO-1 (green), CD31 (red), and DAPI (blue) at 3 days after tMCAO in three groups. The white arrows indicated the gap formation of ZO-1. Bar graph showed the quantification of gap formation of ZO-1. Scale bar = 25 μm. Data are mean ± SD, *N* = 6 per group, ^*^p < 0.05 and ^***^p < 0.001.

Occludin and ZO-1 expression presented a gap in the endothelial cell margin of the cerebral microvessel after ischemic injury. CD31/occludin double staining results showed that OPC transplantation alleviated the disruption of occludin (Figure 4A, *p* < 0.001). CD31/ZO-1 double staining results suggested that OPC transplantation alleviated the disruption of ZO-1 (Figure 4B, *p* < 0.05). OPC transplantation significantly reduced gap formation after tMCAO.

### Inhibition of β-catenin aggravated inflammation and BBB disruption

**Figure 5:**
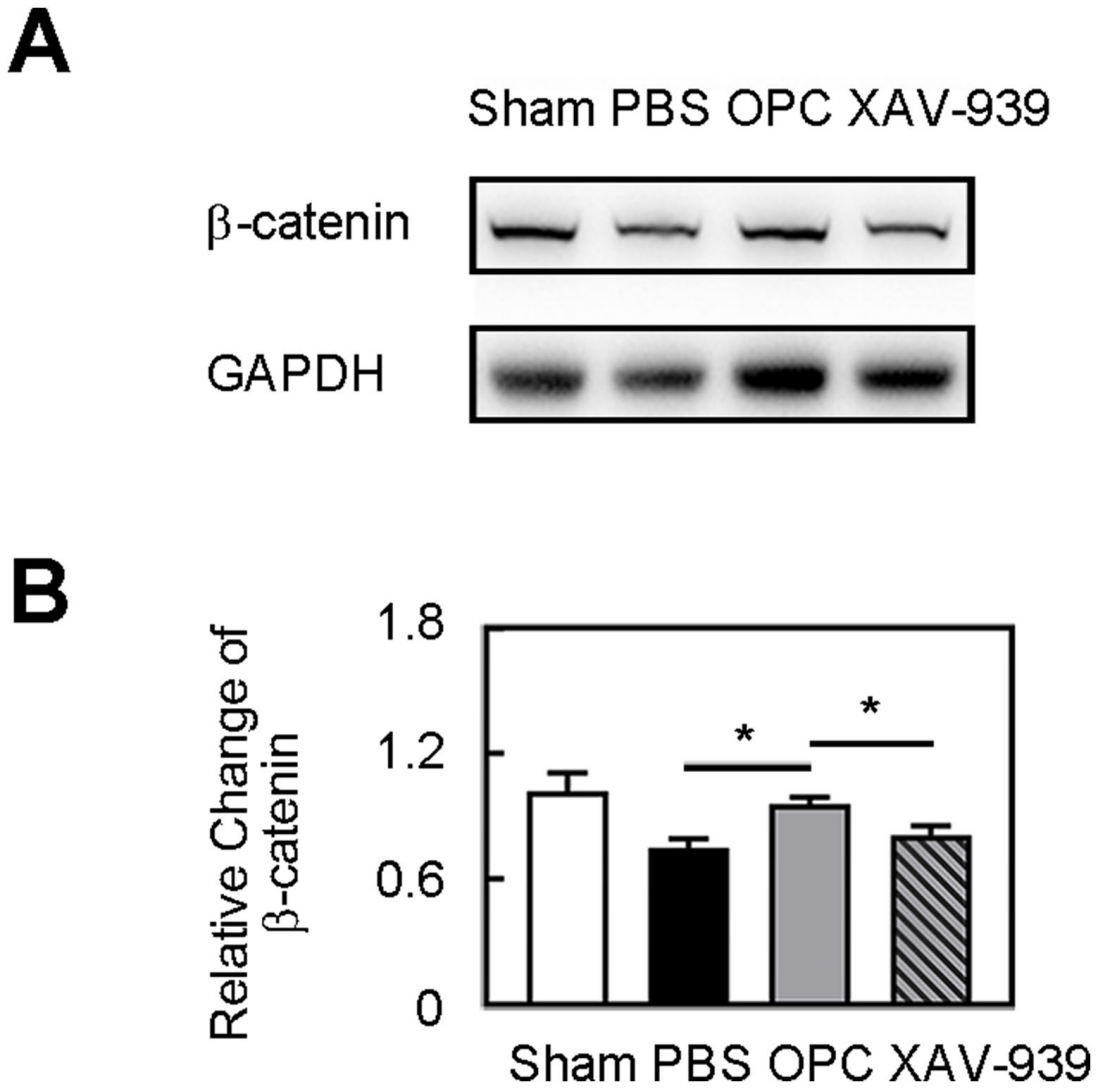
OPC transplantation increased β-catenin level. **(A)** Western blot of β-catenin expression in sham, PBS, OPC, and OPC plus XAV-939 groups at 3 days after tMCAO. **(B)** Bar graph of relative expression of β-catenin. *N* = 4 per group. Data are mean ± SD, ^*^p < 0.05.

**Figure 6:**
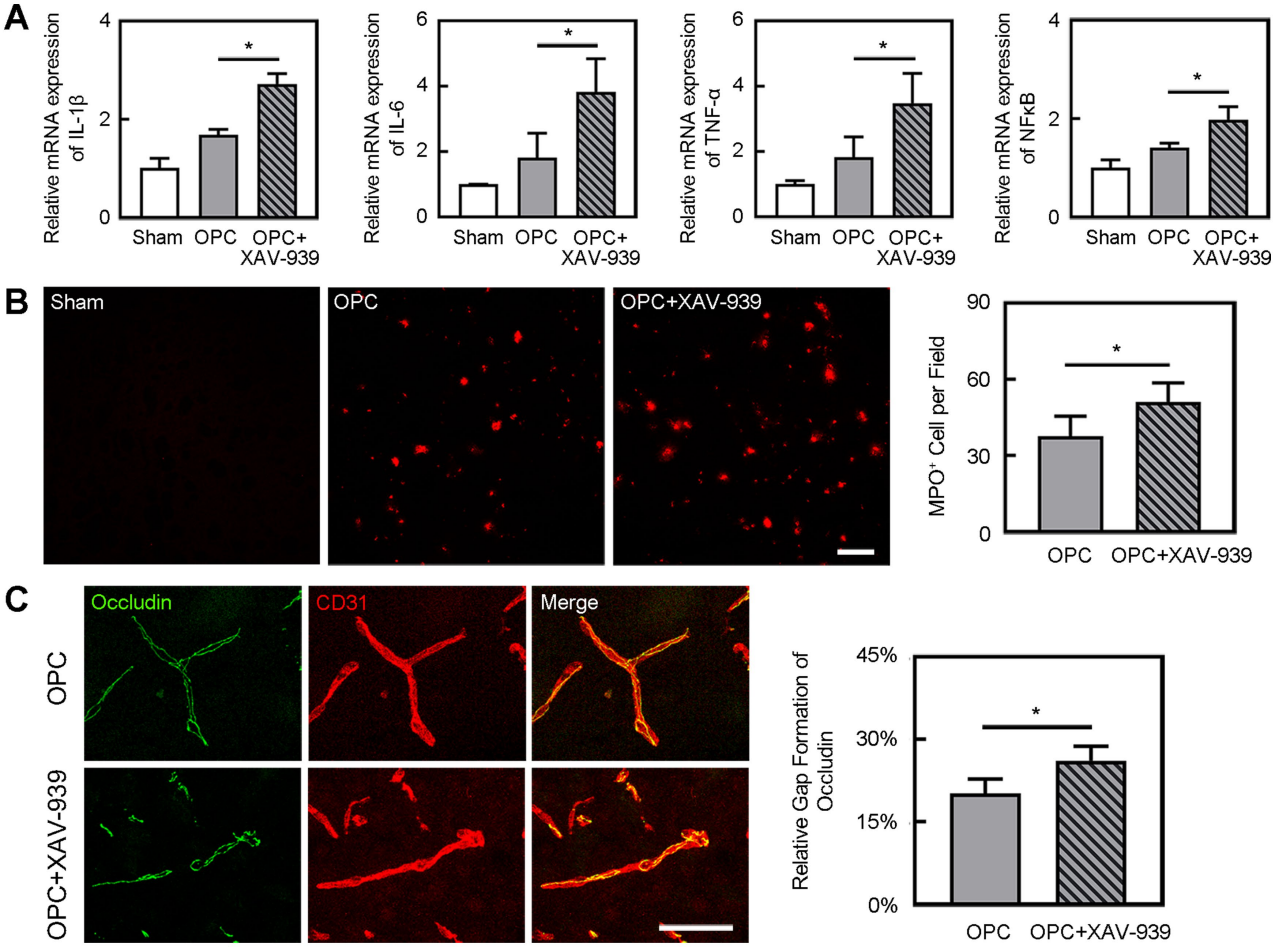
Inhibition of β-catenin aggravated inflammation and BBB disruption. **(A)** Bar graphs showed the mRNA level of IL-1β, IL-6, TNF-α, and NF-κB in the sham, OPC, and OPC plus XAV-939 groups at 3 days after tMCAO. *N* = 4 per group. **(B)** Representative images of MPO staining in the peri-infarct area at 3 days after tMCAO in sham, OPC, and OPC plus XAV-939 groups. Bar graph showed the quantification of MPO^+^ cells. Scale bar = 100 μm. *N* = 6 per group. **(C)** Three-dimension reconstruction confocal microscopy images of occludin (green) and CD31 (red) at 3 days after tMCAO in OPC and OPC plus XAV-939 groups. Bar graph showed the quantification of gap formation of occludin. Scale bar = 50 μm. *N* = 6 per group. Data are mean ± SD, ^*^p < 0.05.

OPC transplantation enhanced β-catenin expression in the ipsilateral hemisphere compared to the control (Figure 5, *p* < 0.05). As we supposed that the downregulated inflammation caused by OPC transplantation was related to the enhanced β-catenin expression, we used XAV-939 to inhibit the β-catenin. The β-catenin inhibitor XAV-939 administration could suppress the β-catenin expression which was enhanced by OPC transplantation (Figure 5, *p* < 0.05). To further examine whether β-catenin was involved in the beneficial role of OPCs after tMCAO, β-catenin inhibitor XAV-939 was injected in OPC-treated ischemic mice. We found that XAV-939 could upregulate the expression of inflammatory factors and increase neutrophil infiltration after tMCAO (Figures 6A,B, *p* < 0.05). Besides, the inhibition of β-catenin downregulated the NF-κB pathway (Figure 6A, *p* < 0.05). The endothelial gap formation was increased after XAV-939 administration (Figure 6C, *p* < 0.05). XAV-939 treatment reversed the protective role of OPC transplantation on the integrity of BBB.

## Discussion

Our study demonstrated that OPC transplantation reduced inflammation, attenuated tight junction disruption of BBB, and improved neurobehavioral outcomes in ischemic mice. The Wnt/β-catenin pathway activated by OPC treatment might contribute to the downregulation of neuroinflammation after ischemic stroke.

Inflammation could exacerbate ischemic tissue damage and worsen clinical outcome in patients with stroke (31). The intense neuroinflammation occurring during the acute phase of stroke is associated with BBB breakdown, neuronal injury, and worse neurological outcomes. Inflammation-driven injury mechanisms in stroke include oxidative stress, increased MMPs production, microglial activation, and infiltration of peripheral immune cells into the ischemic tissue (32). Many types of stem cells can potentially treat ischemic stroke (33). Mesenchymal stem cell therapy could reduce inflammation and attenuate BBB disruption in mice after ischemia (22). Our data showed that OPC transplantation significantly attenuated inflammation after ischemia. Inflammatory cytokines IL-1β, IL-6, and TNF-α and neutrophil infiltration were reduced in OPC-treated mice compared to the control group. The suppression of inflammation of OPC transplantation ameliorated neurobehavioral deficiency.

Inflammatory interactions at the blood-endothelial interface include cytokines, chemokines, adhesion molecules, and white blood cells, which are crucial for the pathogenesis of cerebral infarction (34). Proinflammatory intracellular signaling cascades and transcription factors, for example, NF-κB, ROS, MMPs, and the release of proinflammatory cytokines, especially IL-1β, IL-6, and TNF-α, are associated with BBB dysfunction after stroke (32). Our results showed that OPC transplantation reduced tight junction protein degradation after ischemia. The protective effect on BBB of OPC transplantation could be through the downregulation of inflammation.

The Wnt/β-catenin pathway is involved in cellular proliferation, survival, differentiation, migration, angiogenesis, and vascular maturation (35). Research indicated that enhancing cerebrovascular Wnt/β-catenin activity would offer protection against BBB permeability and neuroinflammation in acute infection (36). Previous studies reported that reactivation of Wnt/β-catenin signaling in vessels during experimental autoimmune encephalomyelitis/multiple sclerosis partially restored functional BBB integrity and limited immune cell infiltration into the brain (37). In our study, OPC transplantation increased the level of β-catenin and downregulated the mRNA level of NF-κB. Furthermore, the inhibition of β-catenin reversed the inflammation inhibition by OPC transplantation and aggravated tight junction protein disruption.

Wnt/β-catenin signaling exerts the anti-inflammatory function partially due to repressing the NF-κB pathway (38). Previous research suggested the negative regulation of NF-κB-mediated inflammatory responses by β-catenin in intestinal epithelial cells (39). Wnt/β-catenin pathway components modulate inflammatory and immune responses via the interaction with NF-κB (40). Our results demonstrated that the level of NF-κB had a negative correlation with that of β-catenin. Therefore, we supposed that OPC transplantation attenuated inflammation by β-catenin and the downstream pathway might be NF-κB.

In our previous study, some transplanted OPCs differentiate into oligodendrocyte which expressed MBP around the myelin at 28 days after tMCAO (17). We found OPCs could secret Wnt7a which activated endothelial cells (16). Besides Wnt7a, OPCs could secret trophic factors, providing trophic signals to neighboring cells. Based on other basic researches on stem cell transplantation, like mesenchymal stem cell and endothelial progenitor cell, we tended to believe that factors released by OPCs were crucial for the beneficial effects of OPC transplantation following ischemic stroke (21, 33, 41).

We previously proved that OPC transplantation’s chronic effects on angiogenesis and remyelination after ischemic stroke. OPC transplantation reduced BBB permeability which indicated the better vascular maturity (17). But we did not focus on the chronic effects on the tight junction proteins in the endothelial cells. The long-term impact needs to be assessed. We ever injected OPCs 7 days after tMCAO and found improved behavior recovery and reduced brain atrophy volume at 28 days after OPC transplantation (18). The delayed OPC transplantation could enhance endogenous oligodendrogenesis, neurite growth, and synaptogenesis.

There were some limitations in our study. Conditional knock-out mice of β-catenin are needed to show a larger certainty of the underlying mechanism. The β-catenin activation in different type of cells in the central nervous system, for example, microglial and astrocyte, should be further explored. A previous study showed that activation of Wnt/β-catenin signaling attenuated ICAM-1/VCAM-1-mediated adhesion of both macrophages and neutrophils to alveolar epithelial cells (42). So other targets need to be studied in future.

In this study, we demonstrated that OPC transplantation attenuated inflammation, which protected BBB, decreased brain infarct volume and improved neurological outcomes after ischemic stroke in mice. This anti-inflammatory function of OPC transplantation might be via activating β-catenin and then affecting NF-κB. OPC transplantation is a promising approach for the ischemic stroke therapy.

## Data Availability

The raw data supporting the conclusions of this article will be made available by the authors, without undue reservation.
